# Improving prehospital and emergency care for patients with mental dysregulation: a comprehensive research agenda

**DOI:** 10.1186/s13049-026-01575-8

**Published:** 2026-02-16

**Authors:** Niek Galenkamp, Geurt van de Glind, Bart Schut, David Baden, Maartje M. J. Singendonk, Lente Werner, Mark van Veen, Lisette Schoonhoven, Floortje E. Scheepers, Wietske H. W. Blom-Ham

**Affiliations:** 1 Institute for Nursing Studies, University of Applied Sciences Utrecht, Utrecht, Netherlands; 2 Person With Lived Experience, Utrecht, Netherlands; 3 Diakonessenhuis Hospital, Utrecht, Netherlands; 4 University Medical Center Utrecht, Utrecht, Netherlands; 5 University of Southampton, Southampton, UK

## Abstract

**Objective:**

To develop a research agenda on mental dysregulation in emergency care settings, that is informed and prioritized by patients' lived experiences, professional expertise from multiple emergency care domains, and the current state of science.

**Methods:**

The Dialogue Model was employed to establish this research agenda. This approach is designed to promote equitable participation of patients and healthcare professionals in the research agenda-setting process. Within this model, a mixed-method approach was conducted, using secondary analysis of qualitative data, an online survey and interviews with key stakeholders, a literature review and a dialogue meeting. Key stakeholders were selected from all domains of the prehospital and emergency care system in the Netherlands, including persons with lived experience, nurses, physicians, mental health professionals and policymakers.

**Results:**

A total of *n* = 29 key stakeholders were involved. A research agenda was developed which comprises two conditional themes (*Understanding the scope and consequences of mental dysregulation in emergency care; Contact between healthcare professional and patient*), three content themes (*Support for emergency care professionals by teams and organizations; Adapting emergency care practices and environments to the needs of patients who experience mental dysregulation; Prevention of dysregulation and hospitalization in emergency care*) and one organizational theme (*Interdisciplinary collaboration around patients who experience mental dysregulation*).

**Conclusions:**

With this research agenda valuable research priorities are provided, acknowledged and prioritized by patients with mental dysregulation and healthcare professionals in the emergency care setting. By involving key stakeholders in the process of developing this research agenda, it covers multiple perspectives and has led to a mutual understanding. As such, it provides a valuable viewpoint for the future direction of research and practice in this field.

**Supplementary Information:**

The online version contains supplementary material available at 10.1186/s13049-026-01575-8.

## Introduction

### Background

Worldwide, the prevalence of people with mental health problems is increasing [1, 2]. This trend is also evident in the Netherlands, where the percentage of the population experiencing mental health problems on an annual basis rose significantly from 17.4% between 2007–2009 to 26.1% between 2019–2022, resulting in a lifetime prevalence of 48.4% [1]. Individuals with diagnosed mental health problems are more susceptible to developing medical conditions [3, 4]. As such, they utilize ambulance [5] and Emergency Department (ED) care more frequently, which increases in line with the severity of their mental health problems [6–8]. The acute onset or exacerbation of somatic illnesses or injuries, combined with the hectic and chaotic environment of the emergency care, can lead to further, or acute, dysregulation. Furthermore, inherent to the emergency care setting, a patient’s history of mental health problems is often unknown upon presentation. This leads healthcare professionals to act on observable symptoms and behavior. Considering this, the term "mental dysregulation", conceptualized as an observable state rather than diagnosed mental health conditions, accurately describes this group of patients [9]. The definition of mental dysregulation can be found in Table 1.

**Table 1:** The definition of mental dysregulation

'A temporary alteration in the mental state that affects communication and interactions with others. It is a state all human beings of all ages can move in and out of when facing conditions that exceed ones means of regulating their responses to these conditions. Related emotions and behaviors can be directed outwardly (examples: raised voice, psychomotor agitation, threatening behavior, property destruction, treatment refusal) as well as inwardly (examples: social withdrawal, inattention, avoidance, depressed mood, emotional numbness, dissociation). This manifests as either Hyperarousal (heightened reactivity) or Hypoarousal (diminished responsiveness) to stimuli and experiences'	

Emergency care professionals find caring for patients experiencing mental dysregulation particularly challenging, often experiencing feelings of frustration and powerlessness [10–12]. Patients face several challenges too, such as problems in interpersonal interactions with staff, noisy environments, and system-level difficulties such as long waiting times and increased length of stay, which may lead to further dysregulation [12–14] They are more likely to encounter stigmatization and diagnostic overshadowing (misattributing physical problems to mental health diagnoses), potentially leading to delayed care or misdiagnosis [15, 16]. Moreover, (re)utilizing emergency care can exacerbate mental dysregulation [17] when actual mental needs are not adequately recognized and addressed. These factors collectively contribute to a complex and challenging situation in emergency care for patients who experience mental dysregulation, which may be disproportionately pressing in rural settings where mental health support is less accessible and patients rely on emergency care professionals who feel ill-equipped to attend to mental dysregulation [18–20].

These challenges, experienced by both patients and professionals in the emergency care setting, urge improvements and better alignment of emergency care for patients with mental dysregulation [12]. However, scientific evidence and adequate interventions are lacking. Research within emergency care is challenging, as patient inclusion, logistics and ethical consideration are demanding [21–23]. Recent literature reviews [24, 25] on existing interventions to improve emergency care for patients who experience mental health problems describe an alarming paucity of coherent, multifaceted and person-centered interventions, that fit within the fast-paced and unpredictable setting as well as the primary goals of emergency care. To advance research and practice in this field, there is a need to synthesize and prioritize relevant research topics, in such a way that evidence is generated relevant to all stakeholders [26].

## Aim

To develop a research agenda on mental dysregulation in emergency care settings that is informed and prioritized by patients' lived experiences, professional expertise from multiple emergency care domains, and the current state of science.

## Methods

### Study design

The Dialogue Model, by Abma and colleagues, was used to guide the development of the research agenda [27]. This model promotes equitable participation of patients and healthcare professionals in the research agenda-setting process. In this approach, a research agenda is developed in six iterative phases: exploration, consultation, prioritization, integration, programming, and implementation. As each phase builds on the previous, some intermediate results are presented in the methods section for readability and to provide insight into how the final result emerged through the process. This study was focused on developing the research agenda, therefore phase 6 (implementation) is not included in this paper. The Dialogue Model acknowledges potential power imbalances between stakeholders and promotes a safe environment for all participants to voice their opinions. Furthermore, it has been successfully applied in various healthcare contexts, including the development of research agendas for specific diseases and patient populations [27].

Within this model, a mixed-methods approach was conducted, using secondary analysis of qualitative data and a review of current literature (exploration phase), an online survey and key stakeholders’ interviews (consultation and prioritization phase), followed by a dialogue meeting (prioritizing, integration and programming phase).

### Study population and setting

The research agenda was developed within the Dutch emergency care system. The project was executed between January 2024 and June 2024.

**Fig. 1:**
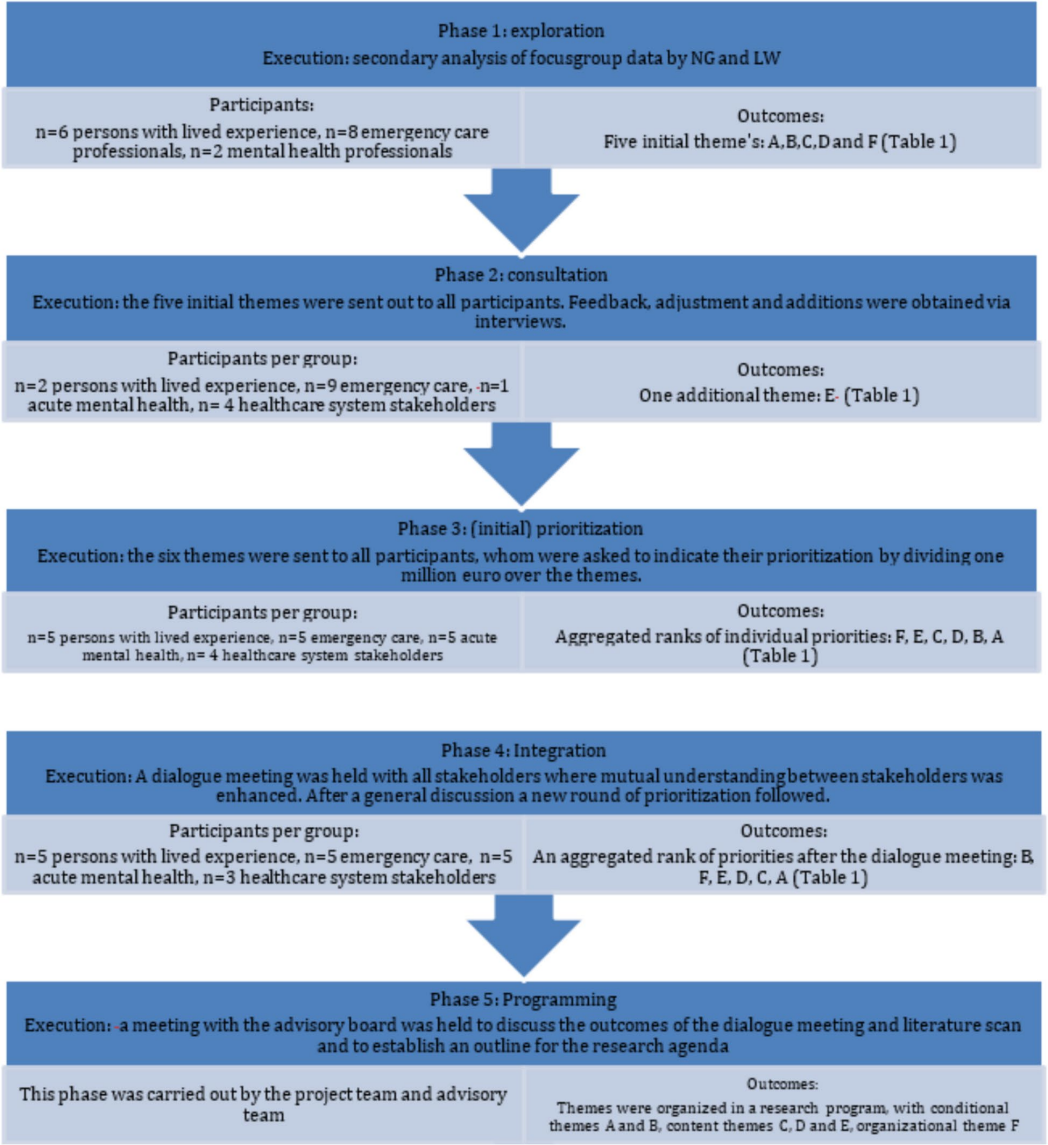
Description of how the first five phases of Abma’s Dialogue model were carried out, including the participants and outcomes per phase

Purposive sampling [28] was employed to include key stakeholders from all domains of the Dutch emergency care system (see Table 2) and patient perspectives for the consultation, prioritization, integration and programming phases. Participants were divided into four groups: persons with lived experience, emergency care professionals (ambulance nurses, emergency care nurses, emergency care physicians), acute mental health nurses and physicians. In the fourth group, healthcare system stakeholders, policymakers, general practitioners (GPs), the national suicide prevention organization and an expert in the domain of policing, safety and healthcare were combined. The number of participants per phase is provided in Fig. 1. See Table 1 for a description of the organization of the Dutch emergency care system.

**Table 2:** Organization of Dutch emergency care

The Dutch emergency care system features three main entry points: GPs, EDs and ambulance (staffed by registered nurses). GPs are the primary gatekeepers for emergency care in the Netherlands [29]. During regular hours, patients can contact their GP for urgent medical needs. Furthermore, emergency number 112 can be called for triage and referral to emergency care services. After hours, patients can access GP services through the Dutch network of 160 GP centres [29]. EDs can be accessed through GP referral, direct transfer by ambulance, or by self-referral [30]. The latter is limited, as GP referral is necessary. Furthermore, acute mental health services are an integral part of the emergency care system in the Netherlands [31]. These are called in by ED or pre-hospital personnel when, besides acute somatic care, acute mental health care is needed. People can also use emergency number 112 for acute mental health needs, however without acute somatic health concerns they will not be referred to the ED. As GPs are the primary entry point for people to enter the mental health care system and acute mental health services are available outside office hours, people rarely visit emergency care services solely for experiencing mental dysregulation. As a result, most patients experiencing mental dysregulation are admitted to the ED due to an acute somatic health problem, with the latter being the main reason for attendance	

### Composition of the team

The research team (NG, LW, GVDG and WBH) consisted of academics with expertise in emergency nursing and mental health nursing and a person with lived experience. The advisory board consisted of a person with lived experience and four professors with expertise in mental health and (emergency) nursing. The advisory board advised on the design of the study. They were updated on the progress of phase 1 to 3, after phase 4 they were involved in the interpretation and translation of the results of phase 4 into the research agenda and a report for the funding organization. Patient and Public Involvement (PPI) is reported in the GRIPP Table 4 [32].

### Study procedure

Below, the execution of the six iterative phases is briefly outlined. The participants per phase and the intermediate results are provided in Fig. 1.

#### Phase 1: exploration

Qualitative data from three focus groups held in 2022 during the PONTAC (Dutch acronym for Mental Dysregulation in Emergency care settings) study were used. The study aimed to improve emergency care for patients experiencing mental dysregulation. In focus groups, areas of improvement were discussed in emergency care for patients who experience mental dysregulation. Two researchers (LW and NG), one of whom is a person with lived experience and the other a social scientist, independently reviewed the data, yielding five initial research themes (A,B,C,D, and F, Table 3).

#### Phase 2: consultation

The initial themes were sent to key stakeholders, who were asked to distribute these within their organization or network and ask for feedback. After they consulted their organization or network, online interviews were held with the stakeholders to gather and discuss the feedback. The consultation demonstrated wide recognition of the initial themes and the addition of one extra theme (E, Prevention, Table 3). Furthermore, several primary research questions were outlined per theme (Appendix A).

#### Phase 3: (initial) prioritization

Stakeholders were asked to prioritize the six themes (including related research questions) by hypothetically dividing 1 million euros among the themes. Results of the prioritization were calculated using the mean sum of money per group, as the number of participants varied per group. Intermediate results from this step can be found in Fig. 1. A detailed description of the outcomes can be found in Appendix B.

#### Phase 4: integration

During the dialogue meeting, the results of the previous step and the results of the literature review were presented. The meeting started with an onboarding activity to increase mutual understanding and sensitize participants to the different perspectives of stakeholders. Thereafter, the themes and the results of the initial prioritization step and literature review were discussed. Subsequently, participants performed the secondary prioritization. The results of this step can be found in Fig. 1 and in more detail in Appendix B.

#### Phase 5: programming

Themes and their priority in practice, as indicated by key stakeholders in phase 4, were weighted and compared with literature review results and discussed with the advisory board, after which the research agenda was composed. A combination of scientific knowledge scarcity and high relevance to practice or science led to an ordering of the themes of research priorities in three categories: conditional, content and organizational (see Table 3).

## Analysis

Qualitative data (interviews and focus groups) were analyzed independently by two researchers, insights were integrated based on consensus. Descriptive statistics of the quantitative prioritization data were analyzed with Microsoft Excel [33].

## Ethics

Participants of the PONTAC focus groups gave consent for the reuse of the data for related research projects (Ethical Committee Research (ECO-GD) of University of Applied Sciences Utrecht number: 198–000–2022). No review of medical/ethical issues was warranted for this project.

## Results

The main outcomes of the study is a research agenda comprising two conditional themes, three content themes and one organizational theme. In total, six central research questions were defined to guide future research to improve emergency care for patients who experience mental dysregulation (Table 3).

**Table 3:** Research agenda themes on mental dysregulation in emergency care settings in the Netherlands, with prioritization ranks from phase 3 (initial prioritization) and phase 4 (integration) (1 highest to 6 lowest priority)

	Theme	Central research question	Initial priority (phase 3)	Priority integration (phase 4)
Conditional Themes	A. Understanding the scope and consequences of mental dysregulation in emergency care	How can mental dysregulation in emergency care be defined and how can its nature and scope be mapped?	6	6
	B. Contact between healthcare professional and patient	How can healthcare professionals be equipped to engage constructively, effectively, and positively with patients with mental dysregulation within the (hectic and unpredictable) infrastructure of emergency care?	5	1
Content Themes	C. Support for emergency care professionals by teams and organizations	What is needed within teams and organizations to better support emergency care professionals in providing emergency care for patients who experience mental dysregulation and to ensure the well-being of these professionals themselves?	3	5
	D. Adapting emergency care practices and environments to the needs of patients who experience mental dysregulation	How can physical and psychological safety be provided to patients who experience mental dysregulation in the emergency care setting?	4	4
	E. Prevention of dysregulation and hospitalization in emergency care	How can dysregulation and frequent re-visits to emergency care be prevented?	2	3
Organizational Theme	F. Interdisciplinary collaboration around patients who experience mental dysregulation	How can healthcare professionals from emergency care, acute psychiatry, general practice, and the police better collaborate around patients who experience mental dysregulation?	1	2

### Conditional themes

#### Theme A: understanding the scope and consequences of mental dysregulation in emergency care

##### Central question

How can mental dysregulation in emergency care be defined and how can its nature and scope be mapped?

##### Background

There is no (quantitative) insight into the scope, nature, and consequences of patients with mental dysregulation in emergency care. This may partly be because primarily somatic complaints are recorded in emergency care [34]. Also, during the initial contact in emergency care the presence of mental dysregulation may be unknown, yet there is still a need for action.

#### Theme B: contact between healthcare professional and patient

##### Central question

How can healthcare professionals be equipped to connect constructively, effectively, and positively with patients with mental dysregulation within the (hectic and unpredictable) setting of emergency care?

##### Background

Secondary analyses of the PONTAC data, results from the dialogue meeting and scientific literature showed a lack of training in how to connect with patients experiencing mental dysregulation [10–12]. This may hamper their ability to positively influence dysregulation while engaging with the patient, which is necessary to prevent escalations. Persons with lived experience, in turn, shared experiences of not being understood or even being stigmatized [16].

### Content themes

#### Theme C: support for emergency care professionals by teams and organizations

##### Central question

What do teams and organizations need to better support emergency care professionals in providing emergency care for patients who experience mental dysregulation and to ensure well-being of these professionals?

##### Background

Caring for patients who experience mental dysregulation in emergency care settings poses significant challenges for teams and organizations in terms of how they can support emergency care professionals. In the PONTAC focus groups, emergency care professionals indicated that they are inadequately prepared and supported in caring for these patients, which is reflected in the literature [10–12]. This leads to frustration and helplessness among emergency care professionals, who often find this care emotionally overwhelming [11, 35].

Throughout focus groups, interviews and the dialogue meeting, it became clear that there is an important role for organizations and teams to adequately equip emergency care professionals to consider this aspect of care as part of their role and to perform accordingly. This should not only be done through education but also by supporting teams in the profound emotional impact of their work in general and with regard to the care of patients who experience mental dysregulation [11, 17] Emergency care professionals emphasized the importance of working together as a team in this type of care, for instance, by being attentive to each other's well-being and shared responsibility for patients.

#### Theme D: adapting emergency care practices and environments to the needs of patients who experience mental dysregulation

##### Central question

How can physical and psychological safety be provided to patients who experience mental dysregulation in the emergency care setting?

##### Background

The focus groups and dialogue meeting indicated that the physical environment of emergency care does not meet the needs of patients who experience mental dysregulation due to factors such as chaos, unpredictability, and brief interactions, which is reflected in the literature. [11, 12, 36, 37] In this hectic and unpredictable environment, ensuring the mental and physical safety of patients is challenging. Beyond the use of restraints and sedation, emergency care offers few strategies to guarantee physical safety, which can result in hospitalization being (re)traumatizing for these patients [11, 17]. Providing psychological and physical safety is essential for ensuring that patients are willing to accept help and is also crucial for patients to regain (parts of) their regulating capacities. The need for appropriate, calm and welcoming emergency care practices and environments was emphasized in the focus groups and dialogue meeting and has been addressed in several reviews [11, 12, 36, 37], however, effective interventions remain scarce.

#### Theme E: prevention of dysregulation and hospitalization in emergency care

##### Central question

How can dysregulation and frequent revisits to emergency care be prevented during emergency care admission?

##### Background

This theme was highlighted and prioritized by both experts and healthcare professionals during the process. Furthermore, adequate follow-up was mentioned as an important factor by people with lived experience in the consultation phase, this barrier is well documented in the literature [12, 38]. There is limited research on how to prevent mental dysregulation in emergency care and how to avoid unnecessary repeat visits for the same issues. During the dialogue meeting, it was discussed that it is inevitable that some people will experience mental dysregulation, also when they require emergency care for somatic health issues. Therefore, this theme focuses on actions and interventions within emergency care that can prevent unnecessary repeat visits. This is regarded tertiary prevention [39, 40]. In the context of this research agenda this may include topics such as: prevention of ‘diagnostic overshadowing’ [15, 41], prevention of psychological trauma during emergency care admission, thorough identification and addressing of underlying psychological issues, and appropriate referral to prevent further harm.

### Organizational theme

#### Theme F: interdisciplinary collaboration around patients who experience mental dysregulation

##### Central question

How can healthcare professionals from emergency care, acute psychiatry, general practice, and the police better collaborate around patients who experience mental dysregulation?

##### Background

During various steps in settings this research agenda, it was stressed that collaboration is suboptimal between the various services involved in emergency care for patients who experience mental dysregulation, resulting in fragmented care. These services include (acute) mental health services, police and general practitioners. This is also reflected in the literature [11, 12]. While the importance of improving care organization is widely recognized by stakeholders, few knowledge gaps were identified within this theme. Field consultations and literature highlight several successful local interventions, but broader implementation is lacking. Successful interventions reported in the literature include the use of multidisciplinary teams and the introduction of mental health liaison teams [42] An overview of effective collaborations and their outcomes, together with targeted research questions to address the remaining gaps in knowledge, can be a valuable starting point for broader implementation.

### Patient and public involvement (PPI)

Following Staniszewska and colleagues [32], in Table 4 presents the Guidance for Reporting Involvement of Patients and the Public 2 – short form (GRIPP-2SF).

**Table 4:** GRIPP 2 – short form

Section and topic	Item
1: Aim	To establish a research agenda that is informed and prioritized by the lived experience perspective.
2: Methods	Persons with lived experience were included in the project team, the advisory board, as co-authors and as participants. Participants were involved in all five phases of Abma’s Dialogue Model addressed in this paper.
3: Study results	The lived experience perspective is an integral, equal and indistinguishable part of each outcome of this study. It was especially important in highlighting the importance of theme B and addressing theme E.
4: Discussion and conclusions	Involving persons with lived experience had a positive effect in each phase of the study. It allowed researchers and involved healthcare professionals to become aware of how patients are impacted by an emergency care admission and interactions with staff, and vice versa. As such, PPI and the methods had a reciprocal effect that led to mutual understanding.
5: Reflections/critical perspective	Future studies in this field could conduct participative observations or include relatives and caregivers to become aware of the perspectives of those patients who are not able to attend to interviews and fill out questionnaires. Overall PPI was highly beneficial for this study.

## Discussion

This research agenda addresses mental dysregulation, a topic that affects a relatively large [43, 44] and growing group [7, 8] of patients who seek emergency care. It offers funding organizations, researchers and healthcare organizations explicit future directions for high-quality research and innovation efforts to improve emergency care for patients who experience mental dysregulation.

The state of emergency care for patients experiencing mental dysregulation has been a pressing issue for decades [45–47]. Although this issue has been prioritized in several emergency care research agendas [48, 49], these broader emergency care research programs lack the required focus to advance the practice of emergency care for the subgroup of patients who experience mental dysregulation. This is the first research agenda that centers this issue and delineates specific future directions for research and innovation for this specific patient group within the context of emergency care. Now it is up to funding organizations, healthcare organizations, patient organizations and research groups to implement the research agenda in their practice. For instance, by funding calls, linking priorities to research groups and setting up new research networks [27]. Dutch funding organization ZonMW^1^ implemented the research agenda in funding calls for nursing and care related research. We will monitor the progress of implementation and have implemented the agenda as a roadmap for our own future research efforts.

It is remarkable that the issues addressed in this research agenda have been consistently identified and documented in the international literature [11, 12, 16, 36], some from the sixties onward [45, 46], indicating that successful innovation on this matter remains lacking on a global scale and underscoring the importance of a comprehensive research agenda on the topic. One possible explanation for the lack of progress is the fact that research in the emergency care context is challenging, in particular enrolling patients from minority groups in studies [50, 51]. A recent review shows that patient and public involvement, beyond participation as research subjects, is underemployed in emergency care related studies [52]. Another explanation may be that interventions are mainly directed towards seeking external expertise, such as psychiatric liaison services [42, 53–55]. While these yield good results, they do not change the practice of emergency care itself. Other interventions focus solely on specific mental health problems, such as the prevention of self-harm [56]. This leads to fragmented interventions, while their content may in fact be beneficial for most patients who experience mental dysregulation, such as adequate follow-up, involvement of relatives and safety planning. Furthermore, interventions developed to address identified issues miss the fit with the unique characteristics of the emergency care setting [24].

To develop comprehensive interventions that are well adjusted to the context in which they will be used, co-creation is considered particularly effective. By involving both patients and healthcare professionals in the development of interventions, these are more likely to meet the needs of both groups in a particular setting [57]. For example, experience based co-design yielded good results for other complex challenges in the emergency care setting, such as aligning emergency care with the needs of patients during palliative care [58, 59]. Co-creating this research agenda with healthcare professionals from emergency care, acute mental healthcare, as well as persons with lived experience, proved effective in identifying future directions within this context and is considered good practice in future research and innovation, as outlined in this research agenda.

Our research agenda revealed two themes that are considered conditional for efforts to succeed in the other themes. First, a clear and comprehensive definition of mental dysregulation in emergency care in both scientific literature and practice is currently lacking [9]. This impedes not only the development of interventions but also prioritization of the issue, adequate measurement of international or national prevalence, its consequences, and comparison of contexts and interventions. Current research on mental dysregulation in emergency care often focuses on specific conditions such as depression [60, 61], delirium, [62–67], substance abuse [68–73], or behaviors like aggression [74–76]. Data from these studies therefore describe only fragmented parts of the concern within emergency care with patients who, in addition to acute somatic complaints, also experience mental dysregulation. These issues are addressed in Theme A, making this theme a prerequisite for conducting high-quality research related to the other themes.

Second, theme B ‘Contact between a patient and a healthcare professional’ was reprioritized as a key priority during the dialogue meeting (phase 4), while it was initially prioritized relatively low in phase 3. This shift in prioritization emphasizes the importance of involving patient representation along with other stakeholders in the field. The dialogue allows for mutual understanding and a different appreciation of what is needed to improve matters for all involved. The major impact of meaningful contact on patients’ well-being was expressed both in the PONTAC focus groups and the dialogue meeting. Furthermore, patients stressed that positive emergency admission outcomes, such as perceived safety and adequate care, are highly dependent on meaningful interactions with staff, which is in line with recent literature [36, 77, 78]. However, the dialogue meeting also highlighted the lack of knowledge and skills among emergency care personnel to establish effective contact with someone experiencing mental dysregulation, this lack is also well documented in reviews of the scientific literature [11, 12]. The major impact of meaningful contact, combined with the major deficit of knowledge and skills, prompted Theme B as crucial and conditional for the success of research on other themes related to mental dysregulation in emergency care.

The content themes are presented as three separate themes (C, D, E), but should be viewed as interrelated. To adequately address the needs of patients and fit with the complexity of the emergency care setting, there is a need to develop multifaceted and integral interventions [24, 25] that cover multiple themes on this agenda. In the development of such interventions, the prevention of further dysregulation and readmission are an important parameters, as indicated in theme E. Key stakeholders indicated the organizational theme (F) on interdisciplinary collaboration as a high priority issue in the practice of emergency care. However, while interventions within the practice of emergency care are lacking, effective interventions have been developed to improve collaboration with mental health services [42, 53–55]. Weighing the practical priorities against the state of the literature led the project team and advisory board to conclude that the issue concerning this theme lies not in a lack of knowledge or interventions, but in the translation of these into practice. Therefore, this issue is programmed as a separate category, with lower priority for the allocation of research funds. Healthcare organizations are urged to take note of this priority concern and adopt available evidence-based interventions.

## Strengths and limitations

Rooting the development of research priorities in qualitative and available data, including insights from emergency care professionals, mental health professionals, academics and persons with lived experience is considered a strength of this study, as it resulted in research topics that are relevant to all groups. The iterative process, including all relevant stakeholders in each phase, following Abma’s Dialogue Model [27], resulted in changing, adjusting and enriching the themes. The variety of methods and interactions in the dialogue meeting led to a process of mutual understanding. As a result, not only the generated themes but also the process of their development yielded important insights for future research and innovation.

Although patient involvement is considered a strength in this study, it should be noted that we only included persons with lived experience who can participate in online surveys, interviews and attend meetings. Not all people who experience mental dysregulation are able to do so [79], for instance when their mental health hinders participation in such activities. This should not be taken for granted. While we argue that this study benefited substantially from the participation of persons with lived experience, we do not claim to represent all perspectives and positions of patients with mental dysregulation in emergency care. It should be noted that there are ways of articulating these ‘silent’ perspectives and positions [79]. In future projects, participatory observations or involvement of relatives or other caregivers of such patients could be a way of representing their perspectives. Another limitation is that the study does not consider whether priorities shift between age groups, future studies are recommended to explore how priorities and needs might shift with age.

Although the research agenda was developed in the Dutch emergency care context, it has global relevance. It builds on international literature [11, 12, 16, 24, 25, 36, 37] and addresses fundamental themes, such as interprofessional collaboration, and core values like safety, communication, and humanity, rather than context-specific issues. By incorporating both professional and patient perspectives, the agenda furthermore reflects universal principles of emergency care, making it adaptable across diverse settings. While it should be noted that rural located emergency care services face specific challenges regarding mental dysregulation, the topics on this agenda align with calls for improvement by professionals and patients in these communities, such as competency building in professionals and improving interprofessional collaboration [18–20]. Future studies should take disparities in rural emergency care for mental dysregulation into account, for example when measuring the prevalence and consequences of mental dysregulation.

## Conclusion

In conclusion, this research agenda provides valuable research priorities as acknowledged and prioritized by key stakeholders, including patients with mental dysregulation and healthcare professionals in the emergency care setting. It contains clear building blocks, including two conditional themes for future research and innovation on this topic, three content themes that cover relevant points of intervention and one theme with relevant directives for organizations in emergency care. By involving key stakeholders in the process of making this research agenda, it covers multiple perspectives and has led to a mutual understanding and viewpoint for the future direction of research and practice in this field.

## Supplementary Information

Supplementary Material 1.

Supplementary Material 2.

## Data Availability

No datasets were generated or analysed during the current study.

## Footnotes

1 The Netherlands Organization for Health Research and Development, a national body supporting health research, innovation, and implementation.
