# Supplementary material for: Improving prehospital and emergency care for patients with mental dysregulation: a comprehensive research agenda
Source: Scand J Trauma Resusc Emerg Med. 2026 Feb 16;34:62. doi: 10.1186/s13049-026-01575-8 (PMC13015176; doi:10.1186/s13049-026-01575-8)
Supplement: Supplementary file 1 — Supplementary Material 1. [file 13049_2026_1575_MOESM1_ESM.pdf]

Appendix A – Research questions derived from the consultation phase

Conditional themes

Theme A: Understanding the scope and consequences of mental dysregulation in emergency care

Research questions based on consultation phase:

- What are the consequences for the patient of an emergency care admission while experiencing mental dysregulation?
- How can the degree of mental dysregulation be effectively triaged and assessed during an acute care admission?
- What are the characteristics (e.g., psychiatric diagnoses, life stage, etc.) within this patient population?
- How frequently do healthcare professionals experience a sense of insecurity when caring for patients with psychological dysregulation, how does this manifest, and what are the underlying causes?
- What types of (physical and mental health) care do patients with mental dysregulation receive before and after admission to emergency care, and what additional insights can be gained by following these patients over an extended period?

Theme B: Contact between healthcare professional and patient

Research questions based on consultation phase:

- Which individuals, other than physicians and nurses working in emergency care, can be engaged to maintain constructive contact with patients experiencing dysregulation?

- How can the knowledge and experience of family members be more effectively utilized, and how can they be supported during the hospitalization of their loved one?
- How can mental dysregulation be de-escalated and normalized within the context of emergency care?
- How can healthcare professionals be equipped to establish constructive, effective, and positive communication with patients experiencing mental dysregulation who come from different cultural backgrounds and/or do not speak the Dutch language?

Content themes

Theme C: Support for emergency care professionals by teams and organizations

Research questions based on consultation phase:

- What are the sources of frustration among healthcare professionals in relation to patients with mental dysregulation, and how can these frustrations be alleviated?
- How can healthcare professionals be equipped to discuss difficult topics such as suicidality and self-harm with patients?
- How can healthcare professionals derive more fulfillment from working with patients experiencing mental dysregulation?
- What training is required to better prepare healthcare professionals for working with this patient population?
- How can healthcare professionals, both individually and as a team, learn from complex case studies involving mental dysregulation?
- What can healthcare professionals learn from other 'high-impact' professions (such as police and military services) regarding the maintenance of mental resilience?

- How can the mental well-being and psychological safety of healthcare professionals be safeguarded when caring for patients experiencing mental dysregulation?

Theme D: Adapting emergency care practices and environment to the needs of patients who experience mental dysregulation

Research questions based on consultation phase:

- How is an admission to emergency care services experienced by patients with mental dysregulation, and how can care and context be adapted accordingly?
- How can a patient with mental dysregulation feel welcomed and safe within the emergency care setting?
- How can a personalized approach for patients with mental dysregulation help reduce dysregulation during an emergency care admission?
- How can the stigma surrounding mental health issues be reduced in emergency care environments?
- How can mutual expectations between healthcare professionals and patients with mental dysregulation be made explicit and managed during hospitalization?

Theme E: Prevention of dysregulation and hospitalization in emergency care

Research questions based on consultation phase:

- How can different organizations collaborate more effectively to share signals of dysregulation and thus prevent escalation?

- How can a patient with mental dysregulation be better assessed so that appropriate (follow-up) care can be implemented, thereby preventing the need for re-admission to emergency care services?
- Can tools such as relapse/prevention/signaling plans help prevent admissions to emergency care services for patients experiencing mental dysregulation?
- What type of follow up care is necessary to prevent future admissions to emergency care services?

Organizational theme

Theme F: Interdisciplinary collaboration around patients who experience mental dysregulation

Research questions based on consultation phase:

- How can the process of somatic clearance (excluding physical causes of mental dysregulation) be restructured so that patients do not have to wait as long?
- How can organizations better share information about a patient, such as during a handover or prior to admission, in a way that does not stigmatize but provides healthcare professionals with clear guidance on what should and should not be done?
- How can professionals from different organizations involved in emergency care for patients with mental dysregulation better understand each other's language, responsibilities, and policies to improve collaboration?
- The primary focus in emergency care is on addressing life-threatening danger and somatic concerns. This is often prioritized over mental health care. How can an integration of both types of care occur, without compromising the primary process, so that patients wait less, are not moved as often, and receive mental health care sooner?

- How can multidisciplinary case discussions help the involved organizations collaborate more effectively?
