# Supplementary material for: Improving prehospital and emergency care for patients with mental dysregulation: a comprehensive research agenda
Source: Scand J Trauma Resusc Emerg Med. 2026 Feb 16;34:62. doi: 10.1186/s13049-026-01575-8 (PMC13015176; doi:10.1186/s13049-026-01575-8)
Supplement: Supplementary file 2 — Supplementary Material 2. [file 13049_2026_1575_MOESM2_ESM.pdf]

Appendix B: intermediate outcomes

Outcomes Step 3: prioritization

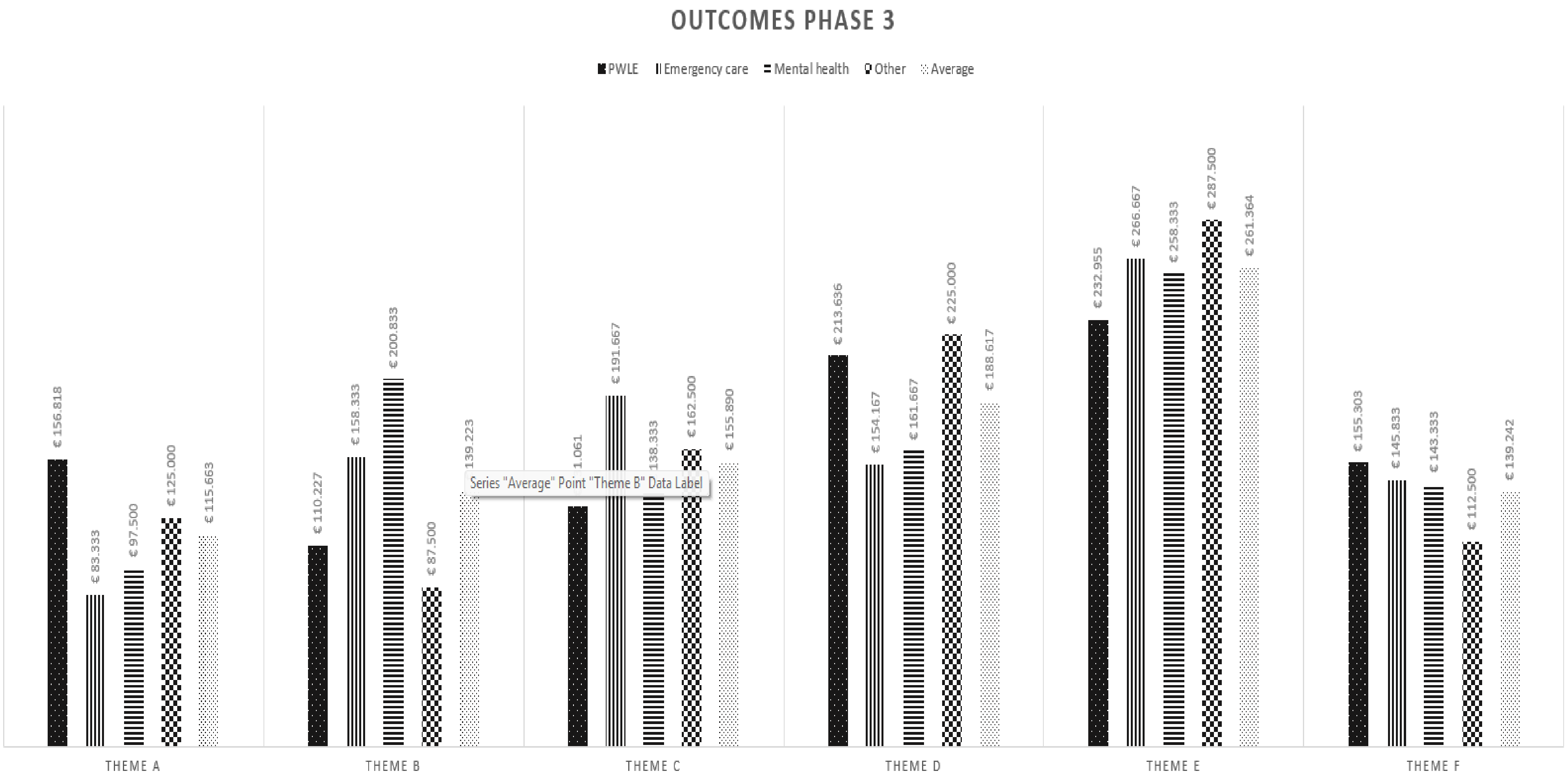

Outcomes Step 4: Integration (Dialogue meeting)

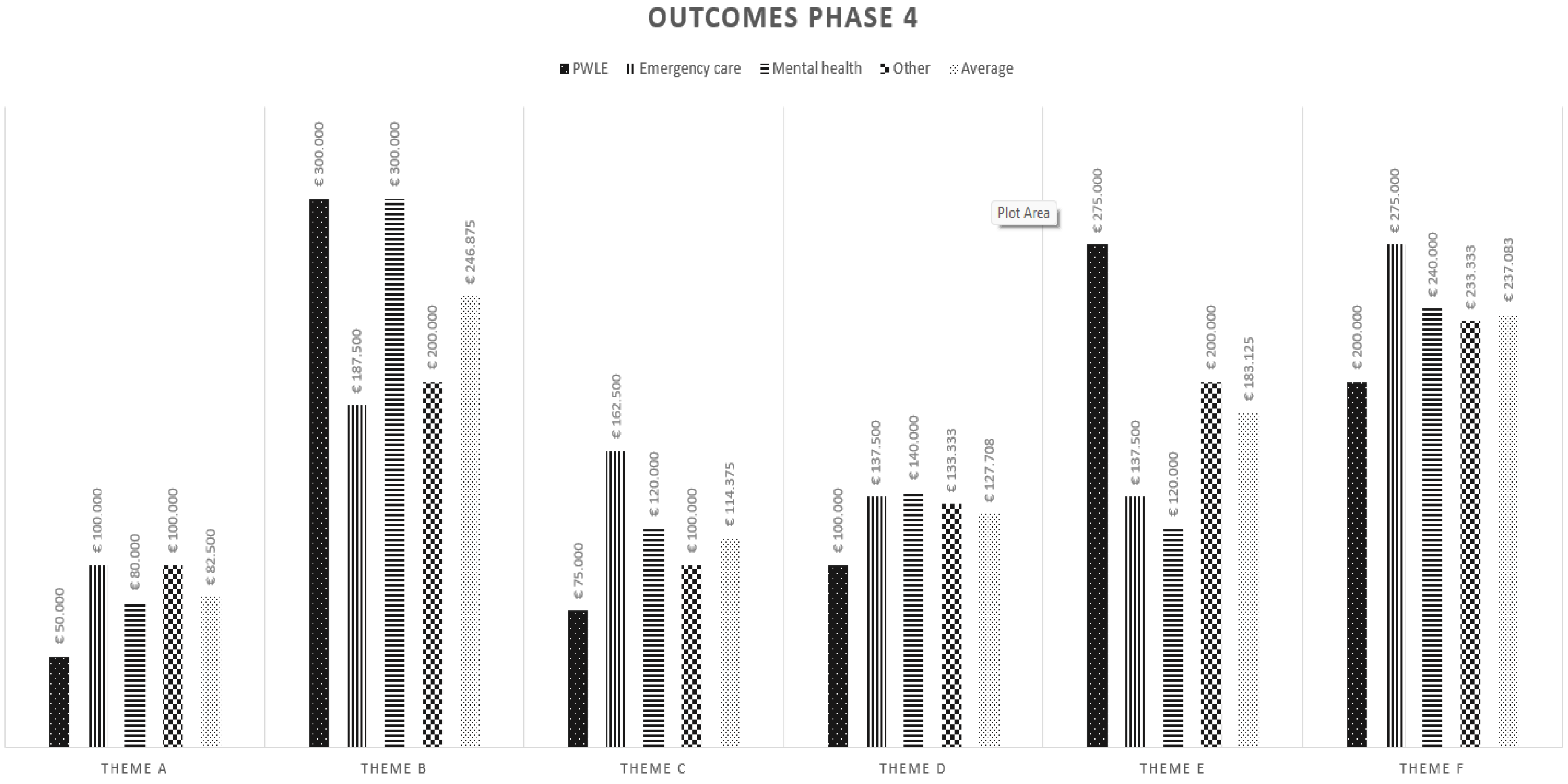

Outcomes Step 4: Integration (Dialogue meeting) Continued

	Average	Persons with lived experience	Emergency careprofessionals (somatic)	Emergency careprofessional (mental health)	Other
**Theme A**	Rank 6 (€74.306)	Rank 6 (€50.000)	Rank 4 - 6 (€122.22)	Rank 6 (€25.000)	Rank 5 - 6 (€100.000)
**Theme B**	Rank 1 (€250.000)	Rank 1 (€300.000)	Rank 2 (€200.000)	Rank 1 (€300.000)	Rank 1 - 2 (€250.000)
**Theme C**	Rank 5 (€117.361)	Rank 5 (€75.000)	Rank 3 (€144.444)	Rank 4 (€150.000)	Rank 5 - 6 (€100.000)
**Theme D**	Rank 4 (€132.639)	Rank 4 (€100.000)	Rank 4 - 6 (€122.22)	Rank 3 (€175.000)	Rank 4 (€133.333)
**Theme E**	Rank 3 (€195.139)	Rank 2 (275.000)	Rank 4 - 6 (€122.22)	Rank 5 (€150.000)	Rank 2 (233.333)
**Theme F**	Rank 2 (€237.500)	Rank 3 (€200.000)	Rank 1 (€266.667)	Rank 2 (€250.000)	Rank 1 - 2 (€250.000)
